# Role of biofilm during groundwater biofiltration of manganese

**DOI:** 10.1038/s41598-025-25228-5

**Published:** 2025-11-21

**Authors:** Jérôme Ducret, Alain Manceau, Christian Lacroix, David Ménard, Catherine Dejoie, Benoit Barbeau

**Affiliations:** 1 Department of Civil, Geological and Mining Engineering, Polytechnique Montreal, 2900 Edouard Montpetit, Montreal, QC H3T 1J4 Canada; 2 European Synchrotron Radiation Facility (ESRF), 38043 Grenoble, France; 3 Laboratoire de Chimie, ENS de Lyon, CNRS, 69342 Lyon, France; 4 Department of Engineering Physics, Polytechnique Montreal, 2900 Edouard Montpetit, Montreal, QC H3T 1J4 Canada

**Keywords:** Water treatment, Manganese removal, Biological filtration, Manganese oxides, Manganese bacteria, Environmental sciences, Microbiology, Water resources

## Abstract

Manganese (Mn) contamination in groundwater poses significant challenges for drinking water treatment. This study explores the mechanisms of Mn removal in a long-term oxygenated groundwater biofilter. The filter media coating primarily consists of abiotic disordered birnessite (δ-MnO_2_) with a microglobular structure and an average oxidation state of approximately 3.45. This material plays a key role in the effective adsorption and oxidation of Mn(II) dissolved in groundwater. The results indicate that Mn removal is predominantly abiotic, with biofilm activity contributing to less than 10% of Mn(II) oxidation. Biological colonization is minimal, as evidenced by the low microbial activity and protein-to-polysaccharide ratio. However, Mn-oxidizing and Mn-reducing bacteria were identified under aerobic conditions, suggesting that they play facultative or complementary roles in Mn cycling. The unexpected coexistence of the two types of bacteria highlights the need for further investigation into their role in δ-MnO_2_ transformation and regeneration. The study provides foundational insights into the dynamics of Mn(II) removal in biofilters and proposes an initial framework for understanding the Mn(II) biogeochemical cycle within such common engineered systems.

**Supplementary Information:**

The online version contains supplementary material available at 10.1038/s41598-025-25228-5.

## Introduction

Manganese (Mn) is a contaminant commonly found in anaerobic groundwater, where it is mostly present as a reduced and dissolved cation (Mn(II)). Lowering Mn concentration in drinking water is a common drinking water treatment objective as elevated Mn concentrations may lead to aesthetic issues (colored water, metallic taste…)^1^ and neurotoxic effects on children^2,3^.

Biological filtration is a commonly approved method for manganese removal, in which oxygenated groundwater is passed through a filter media such as sand to promote biofilm development. Over time, the filtration media becomes progressively coated with a layer of manganese oxides (MnOx) formed through the biogenic oxidation of Mn(II)^4,5^. This oxidation process is partly biotic, facilitated by various microorganisms^6,7^, and occurs predominantly through extracellular mechanisms^8^. These include direct oxidation catalyzed by proteins or polysaccharides, and indirect mechanisms involving changes in local environmental conditions^5^. The process is also partly abiotic, occurring through adsorption onto MnOx surfaces^9^.

Bruins et al.^4^showed that MnOx (later identified as birnessite^9^,) is initially biogenic and gradually evolves toward abiotic precipitation/formation in less than 18 months. Mn removal efficiency by the media progresses over time, as demonstrated by the superior performance of a 15-year-old media compared to a 3-year-old media collected from biofilters^10^. Moreover, it was reported that Mn removal was reduced by 20–40% after eleven years of operation of a full-scale filter^11^.

Although most often described as a biological process^12^, recent studies showed that Mn(II) removal is mainly abiotic^13,14^. In fact, Yang et al.^14^ tested multiples bacterial inhibition methods on a mature media to conclude that short-term Mn(II) removal was not impacted as long as the inactivation method did not alter the MnOx. The use of microbial inhibitors does not necessarily have an impact on the extracellular matrix already formed, which actively participates in the biological oxidation of Mn(II)^5^. Although biological and abiotic Mn(II) oxidation processes are known to be interrelated, maintaining MnOx reactivity over longer time span^15^, it remains important to accurately distinguish between biological and abiotic removal mechanisms within a biofilter in order to properly predict its performance.

The biogeochemical cycle of Mn has been extensively studied in marine environments, with the identification of the coexistence of aerobic and anaerobic bacteria that can respectively oxidize Mn (MnOB)^16^ or reduce Mn (MnRB)^17^ or perform both roles depending on local redox conditions^18^. The co-existence of MnOB and MnRB has been observed in loose deposits from a chlorinated distribution network^19^ but this co-occurrence remains to be confirmed in aerated groundwater biofilters. Such a dynamic could influence treatment performance under shifts in water quality, as has been observed in riverbank filtration systems^20^. Understanding whether such microbial co-existence occur in biofilters is critical for predicting system stability and optimizing operational strategies under varying water quality conditions.

The chemical Leucoberbelin Blue I (LBB) was shown to reduce MnOx to Mn(II)^21^, a characteristic which has been used with success to quantify the Mn oxidation activity of enzymes^22^. We hypothesize that biological Mn oxidation in a biofilter can be quantified after biofilm extraction by adding LBB in the presence or absence of sodium azide (NaN_3_) to discriminate the contributions of the MnOx and the cells present in the biofilm from the extracellular matrix. This is the first study to examine the Mn oxidizing capacity of a biofilm after its extraction from a biofilter media.

This study aims to investigate the role of biofilm on Mn removal by characterizing the biofilter media surface, differentiating the mechanisms of Mn(II) oxidation within the biofilm, and confirming the presence of both MnOB and MnRB in the biofilm.

## Materials and methods

### Description of the water treatment plant

The pressure-driven biological filter under investigation has been in operation for over 20 years and feeds a small community in the southern part of Quebec (Canada), with a mean filtration velocity of 12.5 m/h. A description of the installation and the sampling procedure is presented in Text S1. The influent and effluent characteristics are presented in Table 1. As the influent contains no detectable iron, the sole objective of the treatment process is to remove approximately 0.4 mg/L of manganese, present primarily as Mn(II).

**Table 1:** Characteristics of the biological filter influent and effluent.

Parameters	Influent	Effluent
Turbidity (NTU)	0.23	0.29
pH	7.72	7.61
Temperature (°C)	13.5	15.2
Dissolved oxygen (mg/L)	8.9	7.6
ORP (mV)	323	343
Conductivity (µS/cm)	612	595
Dissolved Mn (mg/L)	0.37	< 0.01
Total Mn (mg/L)	0.39	< 0.01
Dissolved Fe (mg/L)	< 0.03	< 0.03
Total Fe (mg/L)	< 0.03	< 0.03
UVA_254nm_	0.020	0.022
TOC (mg/L)	1.22	1.30
DOC (mg/L)	1.24	1.19
BDOC (mg/L)	0.13	0.13
ATP (pg/mL)	2.56	1.74

### Biofilm characterization

Biofilm can be defined as a microbial community embedded in an extracellular matrix composed of biological products such as extracellular polymeric substances, including MnOx which can be formed by biological oxidation of Mn(II). The biofilm, including its MnOx content, was extracted from the media sample by 3 cycles of 5 min sonication in phosphate buffer according to the method developed by Amini et al.^23^ which was further optimized for our biofilter media samples (Text S3).

**Biochemical characterization:** Proteins were quantified at 560 nm with a micro-plate reader (Tristar2, Berthold Technologies) using the MicroBCA™ protein assay kit (Number 23235, Thermofisher®). Polysaccharides were quantified using the phenol method^24–26^. Metals (Mn, Fe, Ca and Mg) in the extracted biofilm solution were quantified by atomic absorption preceded by acid digestion with concentrated HCl^27^. Mn average oxidation state (AOS) in the extracted biofilm was determined using the LBB method^21,28^. More details about the methods used can be found in Text S4.

**Total/viable cells and diversity:** Flow cytometry (BD Accuri™ C6 Plus, BDbiosciences) after LIVE/DEAD BacLight™ staining was performed in duplicate on the biofilm extraction water to assess bacterial viability^29^. Phenotypic diversity (alpha and beta) were determined from flow cytometry data using the Phenoflow method^30^.

**Bacterial identification:** MnOB and MnRB were isolated by culture using Mn-oxidation^31^ and Mn-reducing^32^ agar media respectively with validation of the oxidation and reduction capacity performed according to the methodology of Cerrato et al.^19^. Pure strain of *Pseudomonas putida* (MnB1, ATCC) and *Shewanella amazonensis* (SB2B, ATCC) were used as positive control for the MnOB and MnRB medium, respectively.

Extraction of genomic DNA was performed on nine MnOB, five MnRB isolates as well as 4 L of raw and filtered water and biofilm extract from the top of biofilter. Sequencing targeted the V4-V5 region of the 16S rRNA genes using the 515FB-926R primers^33^ on an Illumina MiSeq Sequencer (McGill Genome Center, Canada). Sequences were processed using the AmpliconTagger pipeline^34^. More details about the microbial quantification and identification are given in Text S5.

### Biofilm activity

Biofilm activity was quantified using two analytical methods: ATP measurements to assess the general microbial activity and LBB oxidation kinetic to measure biological Mn oxidation activity.

**ATP analysis:** Intracellular ATP quantification was performed on the biofilm extract using the protocol described in Text S2, while extracellular ATP was quantified on the filtrate to check the integrity of the biofilm extracted by sonication.

**Mn oxidizing activity:** LBB reacts specifically with oxidized Mn to form Mn(II), and the number of electrons exchanged is directly proportional to the absorbance of the reactive solution at 630 nm^21^. The following methodology was developed to quantify Mn oxidizing activity of the biofilm extracts.

***Total biofilm oxidizing activity:*** The total Mn(II) oxidation activity of the biofilm (including MnOx) was determined in duplicate by monitoring for 8 h the oxidation of a Mn(II) solution by extracted biofilm (contact times: 0 min, 30 min, 1 h, 2 h, 4 h and 8 h). Absorbance measurements at 630 nm were performed on a solution containing 0.1 mL of the biofilm extract, 0.1 mL of a solution containing 1.5 mM of Mn(II) (MnSO_4_.H_2_O in phosphate buffer) and 0.1 mL of 0.04% (w/v) LBB spiked at each contact times (in different test wells). ***Inhibited microbial oxidizing activity****: S*amples were spiked with NaN_3_ (C_final_ = 0.015M) to reduce the activity of the respiratory chain. Then the LBB solution was spiked in the test wells prior to the 8-h monitoring of absorbances as described above. As LBB rapidly dissolves MnOx, Mn oxidation was assumed to only result from other components of the extracellular matrix. The complete reduction of MnOx by LBB was validated by the absence of changes in the absorbance of controls experiments without MnSO_4_.

For both types of assays, LBB was measured using the analytical method described in Text S4. A summary of the differences between the three types of Mn oxidation activities is presented in Table S1. Influence of the addition of LBB on the biofilm activity is discussed in Text S4.

### Biogenic manganese removal modeling

**Kinetic data modeling*:*** The LBB analysis quantifies electrons exchanged to Mn(II). However, to determine the rate of Mn(II) oxidation by the biofilm, it is necessary to select the number of electrons exchanged during Mn(II) oxidation by the biofilm. In this study, we assumed an average oxidation state (AOS) for biogenic MnOx of 3.5 (3.5 implies the exchange of 1.5 electron for each Mn(II) being oxidized) (Eq. 1**).**

1 $${Mn}^{+2}+{O}_{2}\underset{biofilm}{\to }{{Mn}^{+3.5}O}_{2}$$

Mn(II) oxidation during LBB assays were described by a pseudo-first-order kinetic model according to Eq. 2. The model parameters *f* and *k* were used to compare the samples with total biofilm activity and the residual biofilm activity after inhibition of the respiratory chain (inhibited microbial activity) which corresponds to the residual activity of the extracellular matrix. The parameters *k* and *f* respectively describe the oxidation kinetic (in h^−1^) and the maximum MnOx oxidative capacity (mg Mn oxidized/cm^3^ of media).

2 $$\left[{{Mn}^{+3.5}O}_{2}\right]=f\times \left(1-{e}^{-kt}\right)$$

**Maximal Mn oxidized by the biofilm in a full-scale biofilter:** The maximum amount of Mn(II) that could theoretically be oxidized by the biofilm in our industrial biofilter was estimated using the factor *f*, determined above for different media thickness, which corresponds to the amount of Mn oxidizable by the biofilm per volume of media. Given that this factor was determined at three media depths, the maximum of Mn(II) oxidized in the whole filter was calculated using a weighted average (Eq. 3). This value was compared to the total Mn removed by the biofilter assuming continuous biofilter operation at 12.5 m/h with Mn(II) concentration of 0.37 mg/L in the influent.

3 $$Max\ Mn\left(II\right)\ removed\ by\ biofilm\ (\frac{g\ Mn}{kg\ media})=\sum_{for\ each\ layer}f*\frac{Volume}{media\ mass}$$

In practice, the removal will be limited by the kinetic due to the short contact time available in a biofilter. Assuming a packed bed with negligible dispersion and advection, Eq. (4) was used to estimate the removal by the biofilm while considering kinetic limitation:

4 $$\frac{\left[Mn\left(II\right)\right]}{{\left[Mn\left(II\right)\right]}_{0}}={e}^{-kt}$$

where *k* is the pseudo-first order kinetic parameter determined earlier (h^−1^), and *t* (in h) is the estimated real contact time assuming a filter bed porosity of 45%.

### Characterization of filter media

The metal content (Ca, Fe, Mg and Mn) of the media was determined by atomic absorption spectroscopy using a PinAAcle 900 F spectrometer (PerkinElmer) which was preceded by acid digestion with HCl^27^. The abiotic vs. biotic origin of the media was determined by electron paramagnetic resonance (EPR) at a frequency of 9.6 GHz^35^ according to the reference values proposed by Kim et al.^36^.

The surface of the coating was studied by environmental scanning electron microscopy (ESEM) (Quattro, Thermofisher) coupled to an energy dispersive x-ray spectroscopy (EDS) detector (Ultim-Max, Oxford Instruments). The depth of the media was studied with a scanning electron microscopy (SEM) (FEG-SEM JSM-7600F™, Jeol®) coupled with an EDS detector (X-max™, Oxfort Instruments) using cut, coated and polished media grains.

The mineralogical nature of MnO_x_ was identified by X-ray diffraction at the European Synchrotron Radiation Facility on beamline ID22^37^. Measurements were performed using standard borosilicate glass capillaries in Debye–Scherrer geometry, Si(111) monochromated beam (E = 35 keV), and a Perkin Elmer XRD1611 detector. The detector geometry was calibrated with the LaB_6_ standard. Mn K-edge X-ray absorption spectra were collected at the Canadian Light Source (CLS) synchrotron on beamline IDEAS (08B2-1) as described in Text S6. Data preprocessing and analysis were performed with Athena^38^. Mn AOS in the solids were determined using the Combo Method based on Mn(II), Mn(III) and Mn(IV) proportions^39^ and a Mn reference database^40^.

### Statistical analysis

Comparisons between results at different depths were performed using the Kruskal–Wallis test using R^41^ a p-value < 0.05 was considered as significant.

## Results

### Media characterization and confirmation of abiotic MnOx with biogenic-like growth structures

**Fig. 1:**
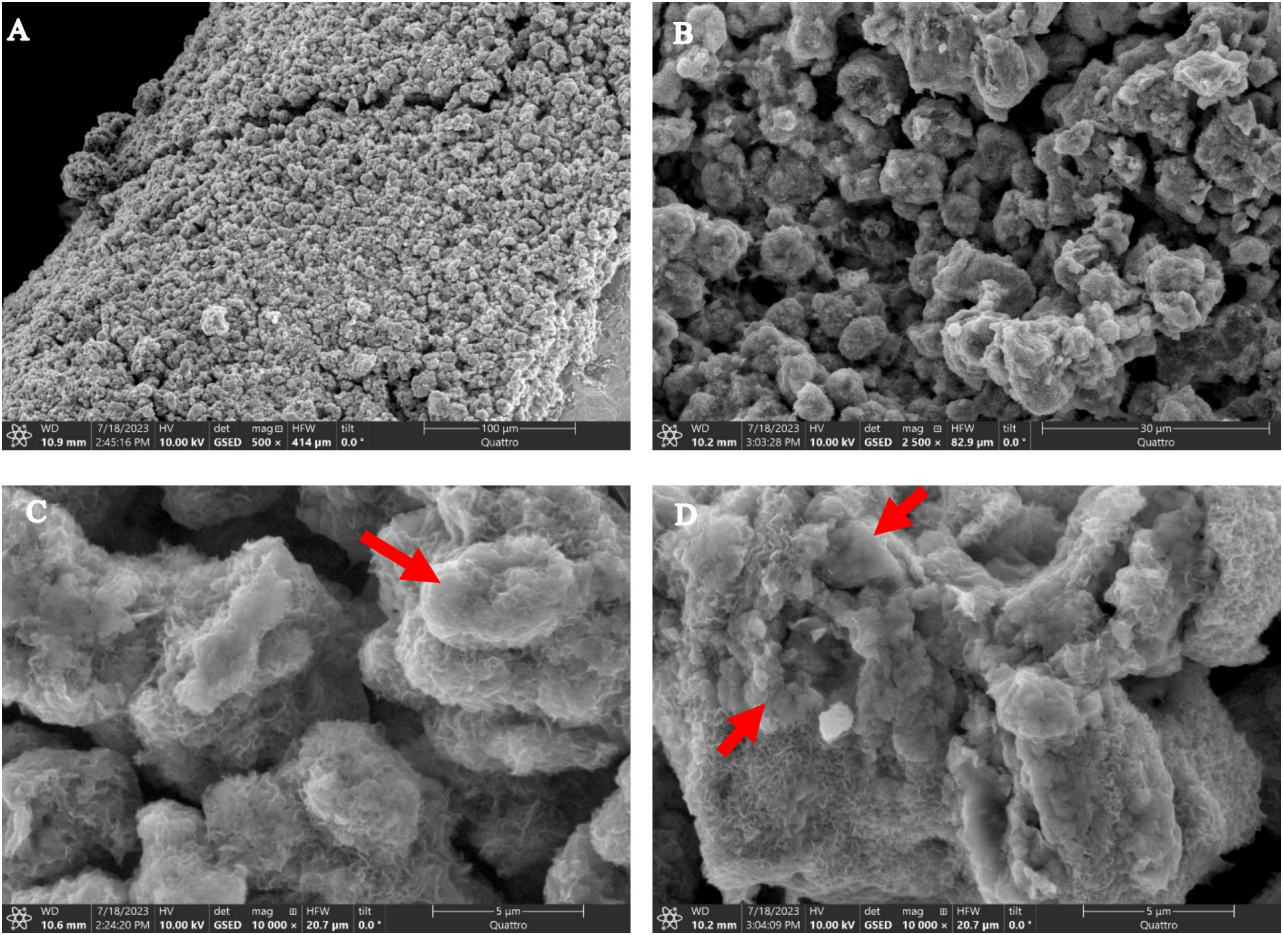
Environmental scanning electron microscopy of media surface at different magnifications (500X for **A**, 2,500X for **B** and 10,000X for **C** and **D**). Arrows point out suspected biological structures.

Figure 1 illustrates the ESEM imaging of the media structure. The media exhibits remarkable homogeneity, characterized by a repetitive globular microstructure resembling coral or sponge-like formations (with Mn and O as major elements), which are typical of abiotic MnOx^4,42^. The presence of overlapping globules, loosely connected to one another, suggests that media growth occurs at specific localized sites. The abiotic origin of the samples from various media depths was confirmed through EPR analysis (Table S2), which showed a minimum linewidth (ΔH of 1870 Gauss (187mT > 1200 Gauss (120mT^36^;). In Fig. 1A and 1B, small particles (indicated by arrows) can be observed directly attached to the globular abiotic MnOx. Under higher magnification (Fig. 1C and 1D), a fluffy structure typical of a layered MnOx becomes visible which as been reported as biotic^4,42^. Although abiotic MnOx dominates the structure, some oxides at the growth centers appear to have a biological origin based on the fluffy structure.

**Fig. 2:**
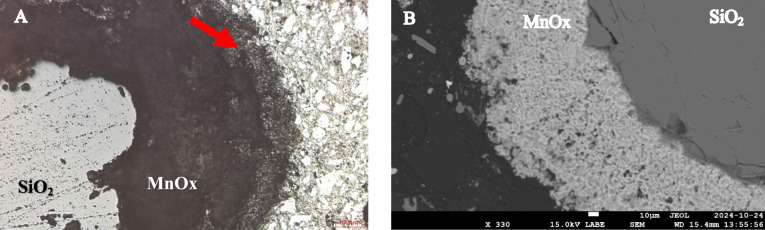
(**A**) Microscopic observation of a cross-section of a filter media grain, with the support media (SiO_2_) in light grey, the MnOx coating in black, and the polymer used for the cross-section in white. (**B**) SEM observation of the filter media cross-section, in black the polymer used to prepare the media grain cross-section, the light grey area corresponds to the MnOx covering of the media and the dark grey area is the SiO_2_ core of the filter media.

The chemical composition of the coating, considering Mn, Fe, Ca, and Mg (Table S2), is over 85% Mn and remains consistent throughout the entire depth of the filter (p > 0.05). A cross-section of a media grain (Fig. 2A) reveals distinct structural features between the surface and the deeper layers of the coating, as well as the presence of interstices, which are further confirmed by SEM imaging (Fig. 2B). The chemical composition of the surface coating analyzed using EDS (with a penetration depth of 0.8 µm in MnO₂ at 10 keV^43^) shows a decrease in the relative Mn content as a function of depth in the biofilter (Fig. S6). This indicates notable changes in the chemical composition between the surface of the coating and the bulk of the filter media. Mn(II) adsorption is more pronounced at the top of the biofilter, as indicated by the lower O/Mn ratios of the bulk coating in the upper section compared to the lower section (Fig. S7).

Because the overall chemical composition of the media did not vary much with depth or after backwashing, only two samples at the top of the biofilter, at the beginning (S1) and at the end (S2) of the biofiltration cycle, were analyzed for Mn AOS using XANES spectroscopy and characterized structurally using XRD and EXAFS spectroscopy. The AOS of Mn is 3.45 ± 0.04 in S1 and 3.56 ± 0.04 in S2 with Mn(III) amounts to 30–35% and Mn(II) amounts to 7–10% (Table S2 and Fig. S8). Mn AOS values are on the low end of those reported previously for a biofiltration pilot system (3.5 and 3.9^9^;). The XRD patterns of both samples are characteristic of turbostratic birnessite (δ-MnO₂) mixed with quartz (Fig. S9), consistent with previous studies^4,9,44,45^. The *d*-spacings of the (100) and (110) reflections are 2.456 Å and 1.419 Å. Their ratio is 1.731, which indicates that the symmetry of the MnO_x_ layer is hexagonal. For both samples, the EXAFS spectra are similar, and their shape is also characteristic of hexagonal phyllomanganate (Fig. S10). Of significance is the shape of the so-called “indicator region” in the 7.5–8.5 Å^−1^ *k* interval^46,47^. When the layer symmetry is hexagonal, the electronic wave is a single antinode and a double antinode when it is orthogonal (Fig. S10.A). Departure of the hexagonal symmetry occurs when the Mn(III) and Mn(IV) cations in the MnO_2_ layer are ordered in rows (Fig. S11). Sample spectra show that the antinode is asymmetric on the left side, which seems to support a small lowering of the layer symmetry. This observation is consistent with the high amount of Mn(III) and Mn(II) cations in the MnO_x_ structure, part of them being probably within the layer. The distortion of the layer structure caused by the Mn(III)/Mn(IV) ordering should be local, because EXAFS is a short-range probe, in contrast to XRD. The emergence of a Mn(III) ordering, together with the low AOS, suggest that the solid gradually transforms into a thermodynamically more stable arrangement over time, such as feitknechtite^48^. This indicates that the coating undergoes very long-term changes.

### Microbial and biochemical characteristics of the extracted biofilm

Table 2 presents the biochemical composition of the extracted biofilm at the beginning and at the end of the biofiltration cycle. Unlike the typical biofilm stratification observed in activated carbon biofilters^49^, the depth-dependent analysis (Table S3) in this study reveals a weak influence of depth on biofilm composition, with only a significant increase in polysaccharide concentration observed with depth (p < 0.05). The biofilm ATP concentration (from 60 to 91 ng/cm^3^) was slightly above values reported by Keithley et al.^50^ for samples collected at the top of two Mn biofilters fed with groundwater (~ 35 ng/cm^3^) but was significantly lower than the ATP concentration reported by McCormick et al.^51^ (~ 250 ng/cm^3^, converted considering a sand density of 2.7) for a Mn biofilter also fed with groundwater. Protein content and the protein-to-polysaccharide ratio in this study were notably low compared to those reported in the literature^50,51^. These low protein levels suggest a cohesive biofilm structure that promotes metal retention^52^, as evidenced by the significant Mn content (22–29 mg/cm^3^) with an AOS of 3.36 and 3.51 confirming the presence of MnOx in the biofilm matrix.

**Table 2:** Biochemical composition of the biofilm in the top of the biofilter at the beginning and at the end of the filtration cycle.

**References**	**This study**		^50^
Parameters	**Begin of cycle**	**End of cycle**	**Top of aerated Mn biofilter**
Total cell counts (log(cell/cm^3^))	7.17 ± 0.02	6.99 ± 0.06	N.R
Intact cell counts (log(cell/cm^3^))	6.31 ± 0.17	6.19 ± 2.77	N.R
ATP intracellular (ng/cm^3^)	90.9 ± 4.6	60.3 ± 7.6	34–35
ATP extracellular (ng/cm^3^)	2.24 ± 1.29	1.43 ± 1.23	N.R
Proteins (mg BSA/cm^3^)	1.72 ± 0.43	1.15 ± 0.51	15.7–15.8
Polysaccharides* (mg D-glucose/cm^3^)	3.11 ± 0.20	2.09 ± 0.18	0.39 and 0.66
Ratio proteins to polysaccharides* (mg BSA/mg D-glucose)	0.55 ± 0.14	0.55 ± 0.25	24 and 41
Biofilm Mn content (mg/cm^3^)	34.5 ± 2.5	25.4 ± 2.9	N.R
Biofilm Fe content (mg/cm^3^)	0.47 ± 0.30	0.49 ± 0.42	N.R
Mn average oxidation state (AOS) in the biofilm	3.36 ± 0.15	3.51 ± 0.10	N.R

Results are expressed by mean ± standard deviation. N.R.: Not reported. * As polysaccharides content are depth related (p < 0.05), the data presented here correspond to the top of the biofilter.

During the filtration cycle, the overall biological activity (lower concentration of polysaccharides and intracellular ATP) of the biofilm decreases significantly (p < 0.05), indicating that backwashing restores the biological activity lost during the filtration cycle.

Fig. S12 shows a comparison of the microbiological diversity present at the top of the biofilter, in the influent and in the effluent. Most bacterial class are less abundant in the top of the filter than in the influent or effluent, except in particular for the Gammaproteobacteria and Alphaproteobacteria which have already been identified as the groups most commonly found in groundwater-fed biofilters^50^. This observation suggests that only a fraction of the influent bacteria can proliferate within the biofilm of the biofilter. The most abundant bacterial genus in the media also belong to the Gammaproteobacteria and Alphaproteobacteria classes, and correspond to the *X35OR*, *Sphingopyxis* and *Coxilla* genus.

Multiple MnOB were isolated by culture, as expected considering the aerobic conditions prevailing in the bioreactor, however unexpectedly multiple MnRB colonies were also isolated from most media samples by aerobic culturing. While several bacterial genera were isolated from MnOB cultures (Table S4), only the *Brevundimonas* genus (found in both MnOB and MnRB cultures, Table S5), was identified in MnRB cultures, along with the *Bacillus* genus, which includes multiple strains previously reported to oxidize manganese^19^. These results highlight the presence of facultative Mn reducers in the biofilter, which could cause Mn release under certain conditions, such as a temporary filter shutdown.

### Limited Mn oxidation by the biofilm extract

**Fig. 3:**
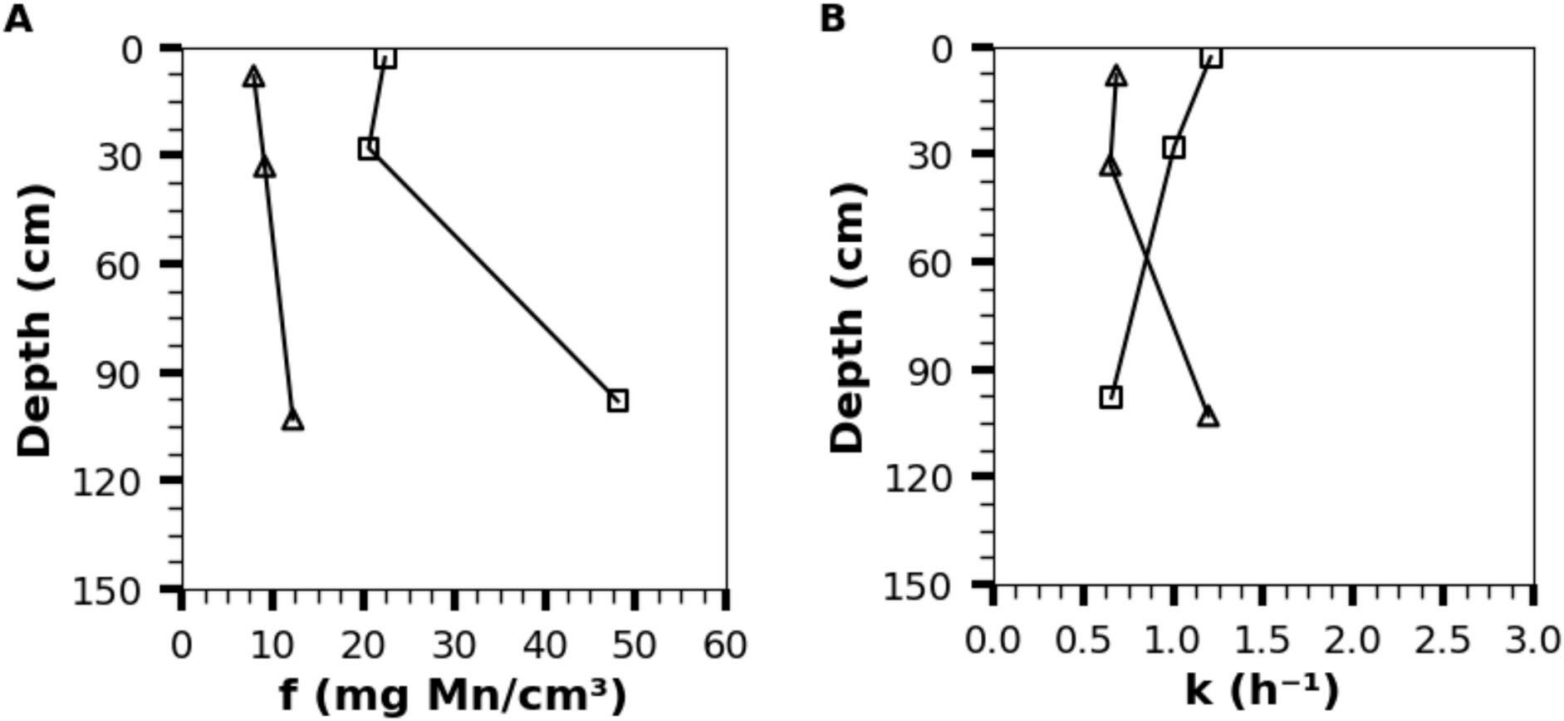
Evolution of the model parameters (Fig. 3A: f, Fig. 3B: k) for Mn oxidation ((□) Total biofilm, (Δ) microbial with inhibition of respiratory chain) in function of depth, at the beginning of the filtration cycle. Fig. S13 presents the same results at the end of the filtration cycle. Fig. S14 presents the raw data of a kinetic assay.

Figure 3 presents the variation of the extracted biofilm oxidative activity parameters (*f* and *k* determined using Eq. 2) in function of filter depth. The total biofilm oxidative activity kinetic constant (*k*) for each depth was used to estimate the biological contribution to Mn(II) removal in the industrial biofilter using Eq. (4). From this calculation, the maximum amount of Mn(II) removed biologically, based ona constant water velocity of 12.5 m/h, is estimated as ≈ 4% of the initial Mn(II) concentration for the biofilter investigated. According to the kinetic study, a minimum contact time of 6 h would be required to remove 99% of the Mn concentration by direct oxidation by the biofilm alone. Mn(II) removal is therefore predominantly abiotic, as confirmed by the study of the elemental composition of the media surface at the top of the filter demonstrating strong adsorption of Mn(II) (Fig. S7). However, once Mn(II) is adsorbed onto the media, it remains available for subsequent oxidation catalyzed by the biofilm or the surface media, independent of water contact time.

The maximum Mn(II) oxidation capacity of the biofilm (factor *f*) was estimated to be approximately 14.5 g Mn/kg of media in the industrial biofilter at the beginning of the cycle. After two weeks of operation, corresponding to the removal of approximately 0.38g Mn/kg of media, a 36% reduction in the maximum capacity (equivalent to 5.2 g Mn/kg) was observed, with the middle section of the biofilter experiencing a more pronounced loss of 47%. The biofilm’s maximum Mn(II) oxidation capacity is sufficient to handle the influent concentration of 0.37 mg Mn(II)/L entering the biofilter. However, under actual operating conditions, the short contact time and competition with other compounds that can also be oxidized by the biofilm prevent the full utilization of this capacity.

The highest Mn oxidation capacity (*f* = 48 mg/cm^3^) was observed in the total biofilm extract (MnOx + bacteria), suggesting that the presence of MnOx and bacteria within the biofilm significantly enhances Mn(II) uptake (p < 0.05). Yet, an average of 36 ± 12% of Mn oxidation capacity persisted after the addition of NaN_3_ and LBB, which corresponds to the oxidation capacity of the extracellular matrix without MnOx. Extracellular polymeric compounds play a non negligible role in the MnOx oxidation by the biofilm especially in the upper layer of the biofilter. Moreover, the oxidation kinetic constant (*k*) was significantly higher (p < 0.05) in the biofilm sample without MnOx compared to the other pathway. This may indicate that the presence of MnOx introduces an additional adsorption step, which increases the overall Mn(II) removal capacity but reduces the apparent oxidation rate.

## Discussion

The kinetic study of biological Mn oxidation predicted that up to 4% of Mn can be directly oxidized biotically when the biofilter operated at a flow rate of 12.5 m/h (according to Eq. (4)). Our approach based on measuring the oxidation rate of extracted biofilm to determine the maximum biological removal yields an estimate which is consistent with those derived using biofilm inactivation techniques without extraction^13,14^. However, the study of biofilm oxidation capacity shows that the contribution of the extracellular matrix is not negligible compared with that of the biofilm and the extracellular activity is not inhibited during biofilm inactivation by NaN_3_. The biofilm present in this biofilter exhibits limited activity but contains a high concentration of polysaccharides, which are known to adsorb Mn(II) and serve as nucleation centers for biological MnOx formation^53^. The presence of such nucleation zones was confirmed by ESEM analysis of the media samples.

The investigation into the biological mechanisms responsible for Mn oxidation showed that the presence of MnOx in the biofilm tends to reduce the biological oxidation rate. MnOx present in the biofilm can be seen as a competitor with extracellular enzymes for reacting with Mn(II). Nevertheless, the total biofilm first-order Mn(II) oxidation rate was 3.4 times higher than the rate we derived for the abiotic oxidation of Mn(II) using biogenic MnOx^54^. This higher reactivity of biotic MnOx is aligned with observations from marine systems^15^.

In the investigated biofilter, the MnOx coating is predominantly abiotic. Although abiotic Mn(II) oxidation is slower than biological oxidation, the prevalence of Mn(III) and loss of hexagonal symmetry in the solid matrix confirm its role in regenerating autocatalytic sites and indicates that MnOx coating continues to evolve over time. Biological regeneration, though minor, helps maintain more reactive higher valency MnOx. Adsorption is the primary Mn(II) removal mechanism, but sufficient dissolved oxygen (DO) is critical for regenerating adsorption sites and ensuring long-term biofilter performance. Future studies should explore the influence of DO on birnessite regeneration.

Biological MnOx oxidation is known to be mostly an extracellular process^8^. In our biofilter, 36% of the capacity is directly related to polysaccharides and proteins which remain in the extracellular matrix and play a major role in the biological Mn oxidation.

Although Mn reducing activity has not been proven in this biofilter, the presence of bacteria with the capacity to reduce Mn (MnRB) may lead to the release of Mn(II) in the biofilter effluent in the event of a prolong change in redox conditions.

The initially biological MnOx coating becomes abiotic over time^4^, and evolves towards a thermodynamically more stable form, which may modify these properties and therefore influence Mn retention over the very long-term. This change, reported for the first time in this study, requires further investigation to understand its implications for the biogeochemical cycle of Mn.

**Fig. 4:**
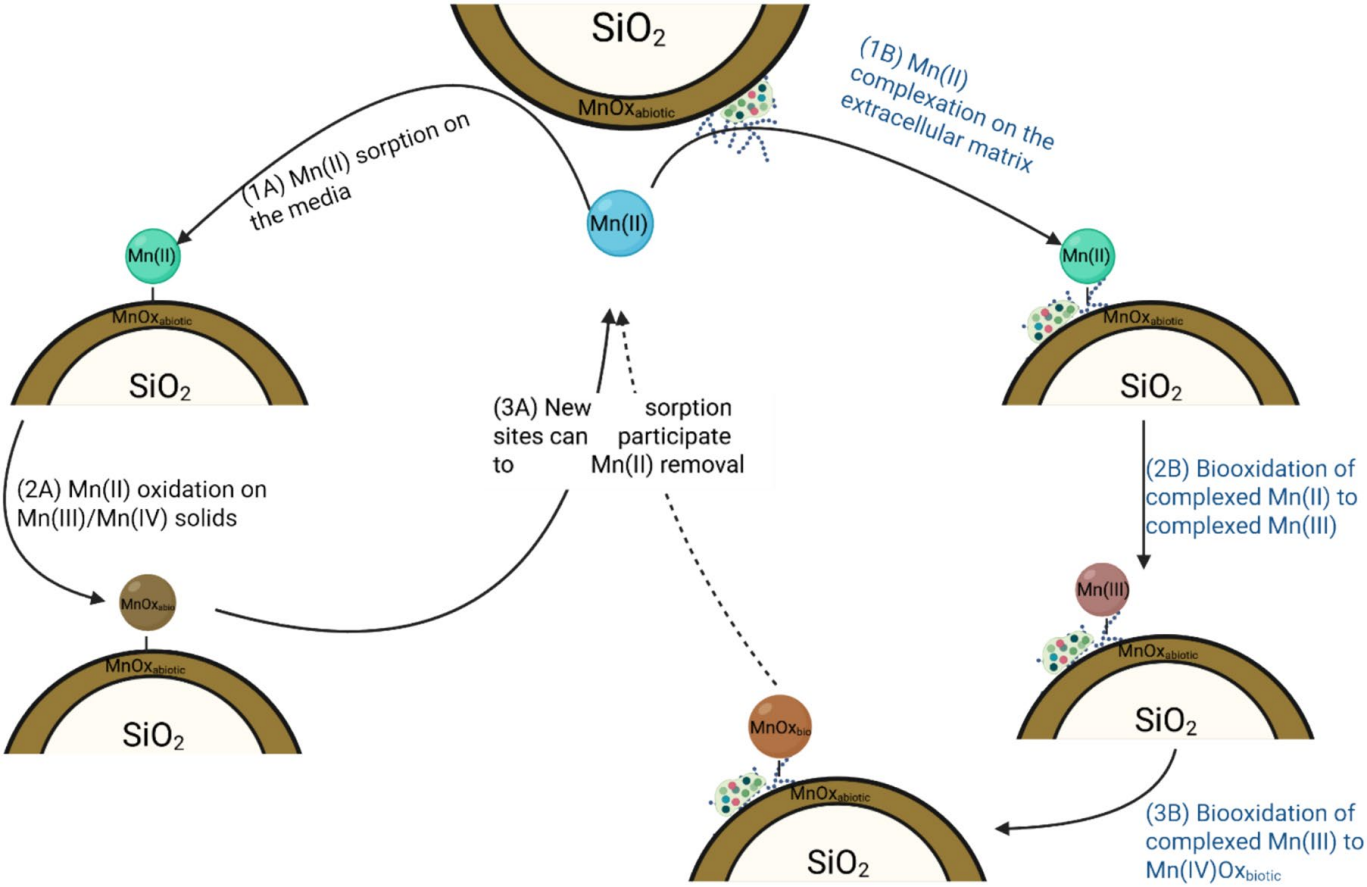
A proposed biogeochemical Mn cycle in a manganese biofilter. The dotted arrows indicate progressive steps. Mn(II) was represented in blue in bulk water, green when sorbed and in brown when oxidized. Created using Biorender.com.

Figure 4 illustrates the proposed biogeochemical cycle of Mn in an aerated biofilter, where Mn uptake during biofiltration is predominantly abiotic. Mn(II) in the liquid phase is primarily adsorbed onto the media surface (step 1 A), with a smaller fraction retained on the biofilm’s extracellular matrix (step 1B). Mn(II) is then oxidized either autocatalytically by MnOx coatings (step 2 A) or bio-catalytically (step 2B, minor contribution). Abiotic Mn(II) oxidation produces solid Mn(III, IV), which is incorporated into the media coating structure^5^. Although the role of oxygen in this step is not fully studied, it likely contributes to regenerating MnOx by accepting electrons during the oxidation of inorganic contaminants^55–57^, consistent with the observed decrease in AOS. Newly formed MnOx sites can then catalyze further Mn(II) adsorption (step 3 A).

Biocatalytic Mn(II) oxidation generates Mn(IV) solids, with dissolved Mn(III) as an intermediate trapped in the biofilm matrix (steps 2B and 3B)^58,59^. Polysaccharides in the biofilm serve as nucleation centers for MnOx, facilitating media growth^60–62^. Biological Mn(II) oxidation is often mediated by extracellular multicopper oxidases (MCOs)^5,58^, although reactive oxygen species may also play a role^15,63,64^.

## Conclusion

This study provides comprehensive insights into the mechanisms responsible for long-term Mn removal in groundwater biofilter. The coating on the filter media was identified as predominantly abiotic disordered birnessite (δ-MnO_2_) with a globular microstructure and an AOS of approximately 3.5. This microglobular structure reflects the progressive growth of abiotic Mn oxides, which are primarily responsible for Mn(II) adsorption and oxidation. The MnOx coating slowly evolves towards a thermodynamically more stable state.

Manganese removal in the investigated biofilter was mainly driven by abiotic processes, with the biofilm contributing less than 10% to the overall Mn(II) oxidation. The biofilter exhibited low biofilm colonization, with limited microbial activity, as evidenced by the low ATP concentrations and low protein-to-polysaccharide ratios. Despite this, the biofilm indirectly supports the regeneration and maintenance of MnOx deposits, facilitating sustained Mn removal performance.

Both MnOB and MnRB were detected under aerobic conditions, suggesting potential facultative behavior or complementary roles in Mn cycling. The presence of facultative MnRB in the biofilter could lead to Mn release in the event of a change in biofilter operating conditions (e.g. long stagnation time, drop in dissolved oxygen, etc.).

Looking ahead, further research is needed to clarify the role of biofilm in the long-term regeneration of filter media, particularly in relation to dissolved oxygen levels. A key question emerges: are microbial communities essential for the sustained functionality and self-regeneration of a mature biofilter, or could abiotic processes alone be sufficient once sufficient coating has been produced? Addressing this question could guide strategies to optimize biofilter performance and ensure its long-term efficacy in Mn removal.

This study emphasizes the predominance of abiotic mechanisms in Mn biofiltration while acknowledging the potentially supportive role of microbial communities in maintaining the long-term reactivity of filter media.

## Supplementary Information

Supplementary Information.

## Data Availability

The data presented in this study are available from the corresponding author upon reasonable request. The raw sequence reads for 16SRNA sequencing are available in the European Nucleotide Archive (ENA) database under project number PRJEB95825 (https:/www.ebi.ac.uk/ena/browser/view/PRJEB95825).
