# Supplementary material for: Role of biofilm during groundwater biofiltration of manganese
Source: Sci Rep. 2025 Nov 21;15:41330. doi: 10.1038/s41598-025-25228-5 (PMC12639001; doi:10.1038/s41598-025-25228-5)
Supplement: Supplementary file 1 — Supplementary Information. [file 41598_2025_25228_MOESM1_ESM.docx]

**Role of biofilm during groundwater biofiltration of manganese**

Jérôme Ducret^1,*^ , Alain Manceau^2,3^, Christian Lacroix^4^, David Ménard^4^, Catherine Dejoie^2^, Benoit Barbeau^1^

^1^: Department of Civil, Geological and Mining Engineering, Polytechnique Montreal, 2900 Edouard Montpetit, Montreal, Quebec H3T 1J4, Canada

^2^European Synchrotron Radiation Facility (ESRF), 38043 Grenoble, France

^3^ENS de Lyon, CNRS, Laboratoire de Chimie, 69342 Lyon, France

^4^: Department of Engineering Physics, Polytechnique Montreal, 2900 Edouard Montpetit, Montreal, Quebec H3T 1J4, Canada

^*^: Corresponding author: Jérôme Ducret, jerome.ducret@polymtl.ca; +33642473622

# Supplementary materials

## Supplementary texts

###### **Text S1**. General characterization of the installation and sampling procedures

The filter (diameter = 3.0 m, initially 150 cm of sand depth, d_10_ =0.95 mm) was started in 2001 and it is fed at a mean water velocity of 12.5 m/h with aerated groundwater supplied from three wells (Fig. S1). According to the operating crew, the biofilter was backwashed once a month from 2001 to 2016 and then twice per month afterwards with an air-water backwash (3 min at 8.2 m/h) followed by a water-only backwash (6 min at 20 m/h).

Water quality was characterized before and after filtration using methods presented in Text S2. The influent and effluent characteristics are presented in Table 1. The initial Mn concentration (0.39 mg/L) was completely removed by the biofilters which operated under favorable conditions for Mn removal (pH 7.7, 8.9 mg O_2_/L, 14^o^C). The absence of consumption of BDOC during filtration indicates a low heterotrophic activity in biofilters which is coherent with the fact that ATP concentration was low and stable, and even slightly reduced in the filter effluent.

After opening the biofilter, we noticed a media height much higher than expected (2 m rather than 1.5m) and compaction reflecting media growth over the years (as presented in Fig. S2). On the surface of the Mn biofilter, a thin rust-colored deposit was observed on top of the media, without strong adhesion, while the actual filtration media remained black in color (Fig. S3).

Biofilter coring was performed in July 2023 at the beginning and at the end of a biofiltration using a 1-inch stainless-steel core drill. The media was then separated in three samples (depths of 0-10 cm, 10-50 cm and 50-150 cm). For each depth range, the media was mixed with a sterilized spatula and sampled in sterile containers.

###### **Text S2.** Water quality analysis

Water samples were collected at the influent and the effluent of the biofilter, just before coring the media. Physicochemical analysis of the water samples were performed onsite, immediately after the sampling using a multiparameter (HQ series, Hach®) for pH, dissolved oxygen, conductivity, a portable turbidimeter (2100Q, Hach®) for turbidity and oxido-reduction potential (ORP) tester (HI98120, Hannah® instruments) for the ORP and temperature. Total and dissolved organic carbon (TOC and DOC) were measured off- site in duplicate using the Persulfate-Ultraviolet method 5310C (American Public Health Association, 2017) with a Sievers M5310C Laboratory TOC Analyzer, before (TOC) and after filtration (DOC) using a 0.45µm supor™ PES membrane (Cytiva®) rinse with 1 L of ultrapure water (Milli-Q integral Water Purification System, Millipore, USA). A filter blank was used to validate the absence of release of organic carbon from the filter. Absorbance UV at 254 nm were measured after filtration using a UV/visible spectrophotometer (Ultrospec 3100 pro) according to method 5910B (American Public Health Association, 2017). Biodegradable dissolved organic carbon (BDOC) was quantified in duplicate after inoculating 125 mL of filtered (0.45µm) sample with autochthonous bacteria (harvested by filtration using a 2.7µm glass fiber filter (Whatman)) and a nutrient solution containing nitrogen and phosphorous (Servais et al., 1989). Biodegradable organic carbon corresponds to the difference between the organic carbon present at time 0 and after 28 days of incubation in dark at room temperature. Total and dissolved Mn and Fe were quantified by atomic absorption (PinAAcle™ 900F, PerkinElmer®) before and after filtration (0.45µm PVDF syringe filter (VWR)), acidified (1% HNO_3_ (trace metal grade, FisherChemical)).

Adenosine triphosphate (ATP) analysis was performed using Quench-Gone™ Aqueous test (LuminUltra®). Briefly, 50mL of sample were filtered using Quench-Gone Syringe Filters (DIS-SFQG), then 1 mL of Ultralyse™ 7 was filtered in the same filter and then harvested in 9 mL of phosphate buffer (pH 7.2, with KH_2_PO_4_ and MgCl_2_, method 9050C) (American Public Health Association, 2017). 0.1 mL of the solution was mix with 0.1 mL of Luminase™ and the ATP was quantified by luminescence using a PhotonMaster™. A blank (only Lumiase™) and a positive (Luminase™ and Ultracheck™ 1) control were performed.

###### **Text S3.** Biofilm detachment from the filter media

Approximatively 5 g of wet filter media were sonicated (three times) in autoclaved glass tube with 25 mL of autoclaved and filtered (0.22 µm, sterile membrane) phosphate buffer in an ultrasonic bath (110W, 40 kHz, model 15337416, Fisher Scientific) during 5 min. The number of sonication cycle was optimized to maximize recovery based on cytometry and ATP analysis (Fig. S4). The 75 mL of phosphate buffer resulting from the three-sonication cycles were then filtered on 40 µm cell-easy strainer (ref 542040, Greiner bio-one) to remove the larger particles which could cause interference. The biofilm extracted solution was characterized the same day that filter coring took place using various methods (see Materials and Methods).

Autoclaved and filtered (0.22µm, sterile MCE membrane, Millex-GS) phosphate buffer was used as dilution water, if needed. For each method a negative control (sterile buffer only) was used.

###### **Text S4**. Biofilm composition analysis

**Polysaccharides:** Briefly, 1 mL of the sample (in glass tubes previously rinsed with 10% v/v trace metal grade HNO_3_) were mixed with 3 mL of concentrate sulphuric acid (trace metal grade, FisherBrand) and 0.6 mL of a 5% (w/v) phenol solution (Anachemia Science). Then the mixture was incubated in a water bath at 90°C during 10 min. The sample was cooled at room temperature during 5 min and the absorbance was read at 490 nm in a spectrophotometer (Ultrospec 3100 pro, 1-cm cell). A 7-points calibration curve using D-(+)-glucose (Sigma Aldrich) was realized 8 points between 0 to 100 mg/L (R^2^=0.9982).

**Extracted biofilm solutions digestion** was performed by mixing 5mL of the sample with 10mL HCl (37%, 3h, 95°C, Fisher Scientific) (Ducret & Barbeau, 2024). Then, metal content was determined by atomic absorption spectroscopy using a PinAAcle 900F spectrometer (PerkinElmer).

**Mn AOS:** A reactive solution of 0.04% (w/v) LBB (Sigma Aldrich) in acetic acid (175mM, Sigma Aldrich) was prepared according to the protocol established by Jones et al. (2019). A volume of 0.1 mL of biofilm extracted solution (diluted if the absorbance read was > 1) was mixed with 0.1 mL of LBB reactive solution and 0.1 mL phosphate buffer. After 30 min of reaction, the absorbance was read at 630 nm on a micro-plate reader (Tristar2, Berthold Technologies). The quadratic calibration curve with seven points (R² = 0.9905) was constructed with triplicate measurements using 0 to 10 µM KMnO₄ solutions (Fisher Scientific) to determine the number of electrons exchanged between MnOx and LBB. The number of electrons exchanged in relation to the Mn concentration in the biofilm is used to determine the AOS of Mn in the biofilm according to equation S4.1.

$$\begin{aligned} Mn AOS=\frac{C_{electron}\left( \frac{mol}{{cm}^{3}} \right)}{C_{Mn}\left( \frac{mol}{{cm}^{3}} \right)}+2 \#\left( S4.1 \right) \end{aligned}$$

**Influence of LBB on biofilm oxidation of Mn(II):** The total biofilm oxidative kinetic constants (measured with punctual spike of LBB on new wells) were significantly lower (p < 0.05) than the values obtained in the permanent presence of LBB (data not shown), which dissolved the MnOx present in the biofilm. Moreover, the addition of NaN_3_, led to a reduction in biofilm oxidation capacity (data not shown). These two results indicate that the addition of LBB had little effect on the biofilm present in the sample, other than dissolving the MnOx present.

###### **Text S5**. Bacterial quantification and identification.

**Total/viable cells:** Before flow cytometry, 300 µL of dilute biofilm extract was mixed with 3 µL of Na-EDTA (500mM) and 3 µL of SYBR Green I or 3 µL of a mix of SYBR Green I and Propidium Iodide for total and viables cells, respectively, incubated during 10 min at 37°C and then analyzed using a flow cytometer (BD Accuri™ C6 Plus, BDbiosciences).

**MnOB isolation:** For each sample, three dilutions (10^-1^, 10^-2^, 10^-3^) were made in duplicate and then incubated aerobically for four weeks at 30°C. A subset of 62 colonies were selected for their variable physiological characteristics (color, shape, etc.), subsequently isolated on a new agar medium and then inoculated in a Mn-oxidation broth media (same composition of the MnOB medium without agar) (Cerrato et al., 2010). After a maximum of two weeks, the oxidation capacity of the isolates was validated in triplicate using the LBB method. If the concentration of MnOx in the culture broth was higher than the concentration of the negative control plus three times its standard deviation, the oxidation capacity of the isolate was considered positive (Cerrato et al., 2010).

**MnRB isolation:** For each sample, three dilutions (10^-1^, 10^-2^, 10^-3^) were incubated in aerobic condition for 4 weeks at 30°C. Similar as for MnOB, a subset of 15 colonies were isolated on a new agar medium and then inoculated in a Mn-reducing broth media (same composition of the MnRB medium without agar) (Cerrato et al., 2010). After a maximum of two weeks, the reducing capacity of the isolate was confirmed by quantifying Mn(II) in the broth medium a after 0.22 µm filtration (PVDF membrane). The absence of MnOx in the filtered sample was validated using the LBB method. If the concentration of Mn(II) (Mn dissolved minus MnOx) in the culture broth was higher than the concentration of the negative control plus three times its standard deviation, the Mn-reducing capacity of the isolate was confirmed (Cerrato et al., 2010).

**Procedure used for sequencing on the 16S rRNA genes:** Genomic DNA was extracted from (i) 9 MnOB, 5 MnRB isolates and from 4 L of raw and filtered water using the FastDNA® Spin Kit (MP Biomedicals) and (ii) biofilm composite solution (10 mL) from media sampled at the top of the biofilter (with DNeasy Powersoil Pro kit, Qiagen).

Beta diversity analysis of the biofilm extracted from various solid samples, based on cytometry, revealed similar phenotypic diversity across the samples (Fig. S5). Therefore, sequencing was performed only on the biofilm from the top of the filter to focus on a representative section. Sequencing targeted the V4-V5 region of the 16S rRNA genes using an Illumina MiSeq Sequencer (McGill Genome Center, Canada) with the 515FB-926R primers (5’- GTGYCAGCMGCCGCGGTAA-3’ and 5’- CCGYCAATTYMTTTRAGTTT-3’) (Parada et al., 2016). Sequences were processed using the AmpliconTagger pipeline (Tremblay & Yergeau, 2019). Each MnOB or MnRB sequence was composed of a single amplicon sequence variant (ASV) representing at least 70% of the sequences read, confirming that these were indeed pure strains. Associated ASVs were subsequently identified with the Standard Nucleotide BLAST using the core nucleotide database (core_nt) (Altschul et al., 1990).

###### **Text S6**. Description of the Mn K-edge X-ray absorption spectra acquisition

Dry filters (top of the filter before and after backwash) were grounded and then loaded into a PTFE sample holder sealed with Kapton tape. The beamline was equipped with a Ge(220) monochromator combined with a Ketek AXAS-M (M5T1T10-H80-ML5BEV) silicon drift detector operating in fluorescence-yield detection mode. The incident energy was calibrated by setting the maximum of the first-derivative of the X-ray absorption near edge structure (XANES) of a Mn(0) foil at 6539.0 eV. Spectra of a Mn(0) foil reference were collected in parallel to those of the samples to verify the stability of the energy calibration during measurements. XANES spectra were recorded over the Mn K-edge region from -200 to -30 eV (step size 10eV, dwell time 2s), -30 to +40eV (step size 0.5eV, dwell time 2s) while extended X-ray absorption fine structure (EXAFS) spectra were collected with a *k*-step resolution of 0.05 Å^-1^. Two scans were collected per sample to increase signal-to-noise ratio.

## Supplementary tables

###### **Table S1.** Description of the experimental differences that enabled us to discriminate between three biological Mn oxidation pathways.

| **Biological Mn oxidation studied** | **Description** | **Concentration of NaN_3_ (M)** | **Addition of LBB** | **Explanation** |
| --- | --- | --- | --- | --- |
| Total | MnOx + microbiological + EPS | 0 | Spiked after incubation (before measurement) | Until the LBB is added, the MnOx present in the solution can participate in the oxidation of Mn(II) |
| Microbiological with inhibition of the respiratory chain | EPS | 0.015 | Spiked before incubation | By adding LBB at the start of the incubation, MnOx present in the biofilm extract is reduced to Mn(II) + Addition of NaN_3_ inhibits the respiratory chain |

*EPS: Extracellular Polymeric substances*

###### **Table S2.** Chemical composition of the coating, electron paramagnetic resonance (9.6 GHz) of the biofilter media as a function of depth, before and after the backwash, XANES results.

|  | **Depth** | **Chemical content (mg element/cm^3^ of media)** | | | | **EPR analysis** | | | **XANES analysis** | | | |
| --- | --- | --- | --- | --- | --- | --- | --- | --- | --- | --- | --- | --- |
|  |  | **Mn** | **Ca** | **Fe** | **Mg** | **H_res_ (G)** | **g factor** | **ΔH (G)** | **Mn AOS** | **%Mn**  **(IV)** | **%Mn**  **(III)** | **%**  **Mn(II)** |
| **Beginning of the cycle** | **0-10**  **cm (S1)** | 239±27 | 27.0±0.8 | 4.7±1.2 | 1.3±0.1 | 2631 | 2.56 | 2221 | 3.45 | 55 | 35 | 10 |
|  | **10-50**  **cm** | 212±20 | 24.3±0.8 | 2.1±0.0 | 1.2±0.1 | 2771 | 2.43 | 2514 | N.D. | N.D. | N.D. | N.D. |
|  | **50-100**  **cm** | 199±14 | 25.4±2.3 | 1.8±0.1 | 2.2±0.3 | 2815 | 2.39 | 2119 | N.D. | N.D. | N.D. | N.D. |
| **End of the cycle** | **0-10**  **cm (S2)** | 206±3 | 27.6±1.3 | 3.7±0.9 | 2.2±0.1 | 2657 | 2.54 | 2542 | 3.56 | 63 | 30 | 7 |
|  | **10-50**  **cm** | 200±36 | 23.8±2.2 | 1.9±0.5 | 1.2±0.2 | 2981 | 2.26 | 1874 | N.D. | N.D. | N.D. | N.D. |
|  | **50-100**  **cm** | 166±9 | 23.7±0.8 | 2.9±0.5 | 1.6±0.4 | 2000 | 3.35 | 3444 | N.D. | N.D. | N.D. | N.D. |

*Chemical content is expressed as mean ± standard deviation. N.D.: not determined.* The accuracy on AOS is estimated to 0.04 v.u.

###### **Table S3.** Biochemical composition of the biofilm in function of depth, at the beginning and the end of the filtration cycle.

|  | **Beginning of the filtration cycle** | | | **End of the filtration cycle** | | |
| --- | --- | --- | --- | --- | --- | --- |
| **Depth** | **0-10cm** | **10-50cm** | **50-100cm** | **0-10cm** | **10-50cm** | **50-100cm** |
| **Total cell counts**  **(log(cell/cm^3^))** | 7.17±0.02 | 7.16±0.03 | 7.17±0.02 | 7.02±0.05 | 6.92±0.01 | 7.03±0.01 |
| **Intact cell counts**  **(log(cell/cm^3^))** | 6.47±0.25 | 6.23±0.07 | 6.24±0.07 | 6.21±0.09 | 6.19±0.02 | 6.19±0.03 |
| **Proteins**  **(mg BSA/cm^3^)** | 1.82±0.39 | 1.86±0.67 | 1.48±0.4 | 1.22±0.87 | 0.81±0.36 | 1.42±0.14 |
| **Polysaccharides**  **(mg D-glucose/cm^3^)** | 3.11±0.18 | 3.33±0.14 | 3.40±0.08 | 2.09±0.20 | 2.20±0.02 | 2.80±0.02 |
| **Ratio proteins to polysaccharides (mg BSA/ mg D-glucose)** | 0.59±0.13 | 0.56±0.20 | 0.44±0.12 | 0.58±0.42 | 0.37±0.16 | 0.51±0.05 |
| **Biofilm Mn content**  **(mg/cm^3^)** | 31.67 | 36.21 | 35.70 | 23.53 | 23.87 | 28.72 |
| **Biofilm Fe content**  **(mg/cm^3^)** | 0.81 | 0.31 | 0.27 | 0.98 | 0.18 | 0.33 |
| **Mn average oxidation state in biofilm** | 3.28 | 3.26 | 3.53 | 3.63 | 3.45 | 3.46 |
| **ATP intracellular**  **(ng/cm^3^)** | 86.9 | 89.8 | 95.9 | 55.6 | 56.1 | 69.0 |
| **ATP extracellular**  **(ng/cm^3^)** | 0.8 | 3.3 | 2.7 | 0.3 | 2.7 | 1.3 |

*Results are expressed by mean ± standard deviation.*

###### **Table S4.** Description of pure MnOB strains isolated by culture.

| **Order** | **Family** | **Genus** | **Colony color** | **MnO_2_ in the liquid culture (µg/L)** |
| --- | --- | --- | --- | --- |
| *Rhizobiales* | *Unclassified* | *Unclassified* | Translucid | 255.5±11.1 |
| *Burkholderiales* | *Comamondaceae* | *Variovorax* | Beige | 134.0±1.1 |
| *Burkholderiales* | *Comamondaceae* | *Variovorax* | Pink | 130.1±2.2 |
| *Burkholderiales* | *Comamondaceae* | *Unclassified* | Pink | 229.7±18.8 |
| *Burkholderiales* | *Comamondaceae* | *Unclassified* | Beige | 244.6±15.5 |
| *Burkholderiales* | *Comamondaceae* | *Acidovorax* | Pink | 168.5±3.3 |
| *Burkholderiales* | *Comamondaceae* | *Acidovorax* | Beige | 121.5±1.1 |
| *Caulobacterales* | *Caulobacteraceae* | *Brevundimonas* | White orange | 125.4±4.4 |

###### **Table S5.** Description of pure MnRB strains isolated by culture.

| **Order** | **Family** | **Genus** | **Colony color** | **Mn(II) in the liquid culture (mg/L)** |
| --- | --- | --- | --- | --- |
| *Caulobacterales* | *Caulobacteraceae* | *Brevundimonas* | Dark brown | 3.97 |
| *Bacillales* | *Bacillaceae* | *Bacillus* | White yellow | 8.06 |
| *Bacillales* | *Bacillaceae* | *Bacillus* | White | 6.61 |
| *Bacillales* | *Bacillaceae* | *Bacillus* | White | 19.06 |
| *Bacillales* | *Bacillaceae* | *Bacillus* | White | 5.57 |
| *Bacillales* | *Bacillaceae* | *Bacillus* | White | 6.79 |

## Supplementary figures


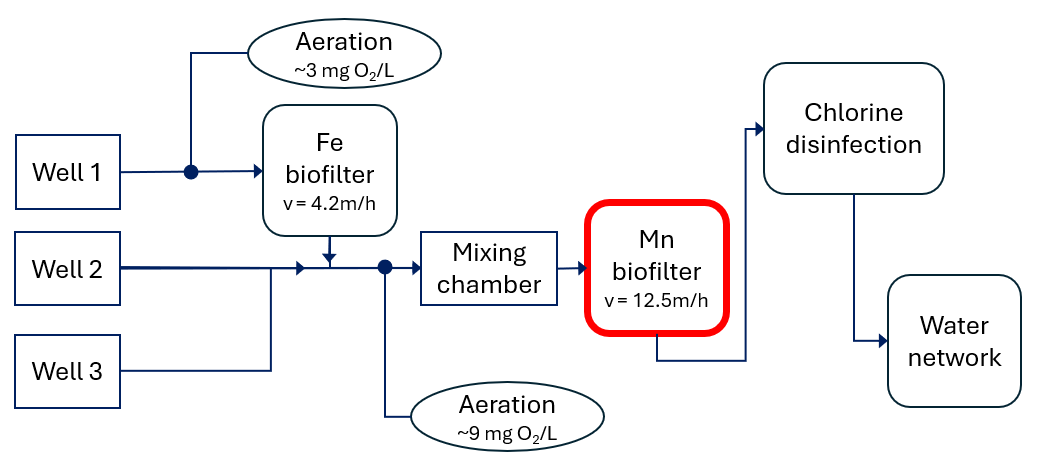


###### **Figure S1.** Presentation of the water treatment plant of the municipality. The Mn biofilter studied is highlighted in red.


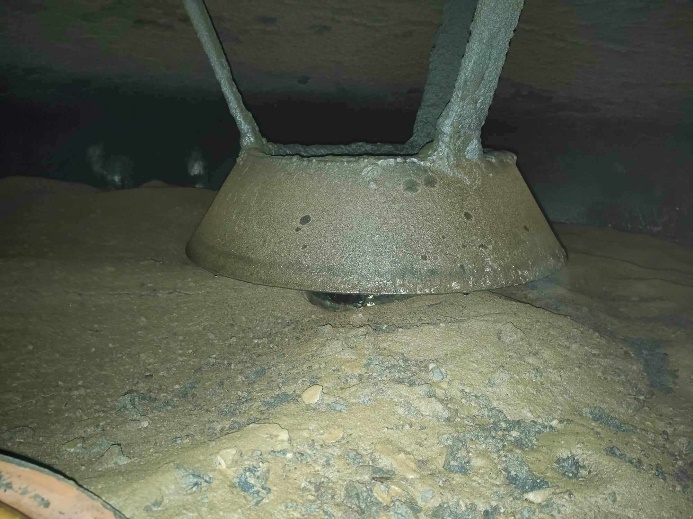


###### **Figure S2.** Photo of the top of the cored biofilter. The media is close to the water distributor and that a thin rust-colored layer covers the top of the media.

*
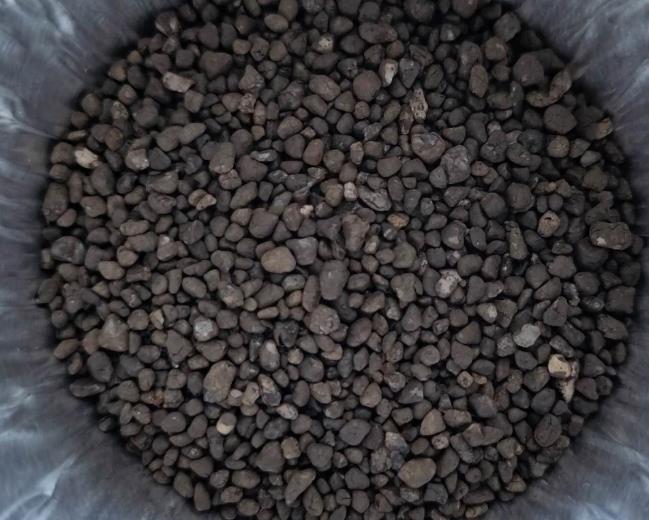
*

###### **Figure S3.** Photo of a media sample taken from the top of the cored biofilter

| 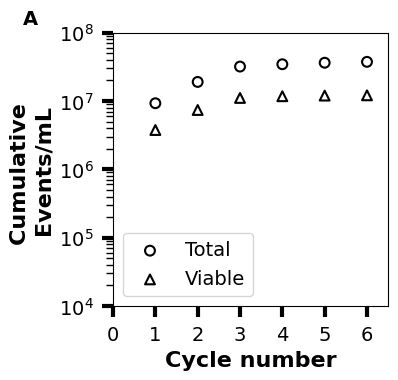 | 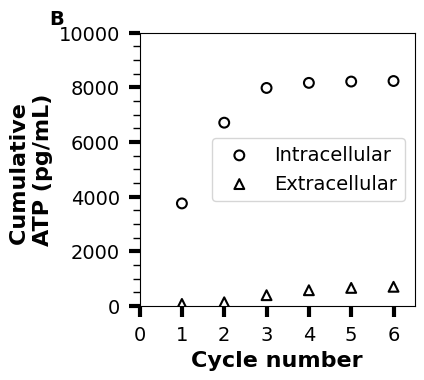 |
| --- | --- |

###### **Figure S4.** Evolution of the cumulative total and viables events (A) or intracellular and extracellular ATP concentration (B) in the 25mL phosphate buffer solution after a 5min sonication cycle. An increase in extracellular ATP is noted from the 3rd cycle onwards, indicating that some of the extracted cells are beginning to be damaged.


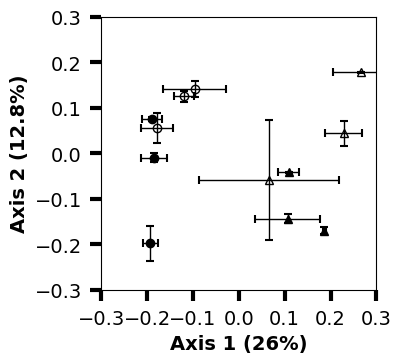


###### **Figure S5.** Evaluation of the phenotype beta-diversity by PCoA realized on flow cytometry data. (●) viable, (▲) total, before (plain symbols) and after (empty symbols) backwash.


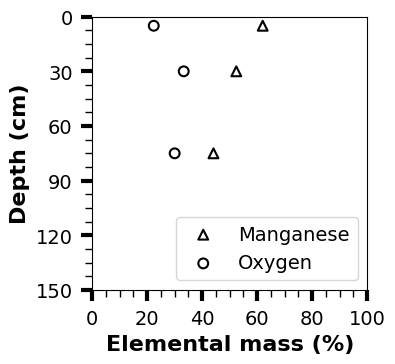


###### **Figure S6.** Evolution of the composition of the surface (EDS penetration depth in MnO_2_ at 10 keV is 0.8µm (Kanaya & Okayama, 1972)) by EDS analysis for Mn and Oxygen.


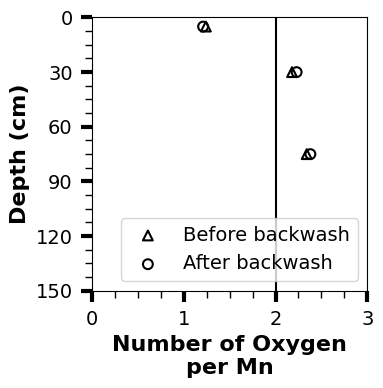


###### **Figure S7.** Evolution of the number of oxygen atom per Mn atom on the surface before and after backwash (EDS penetration depth in MnO_2_ at 10 keV is 0.8µm (Kanaya & Okayama, 1972)). The vertical line represents the number of oxygen atom per Mn atom for the whole coating determined by EDS of a cross-section of the media coating

| 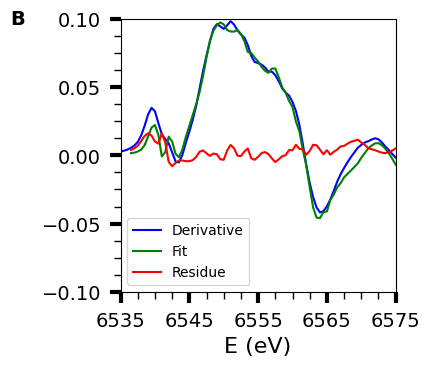 | 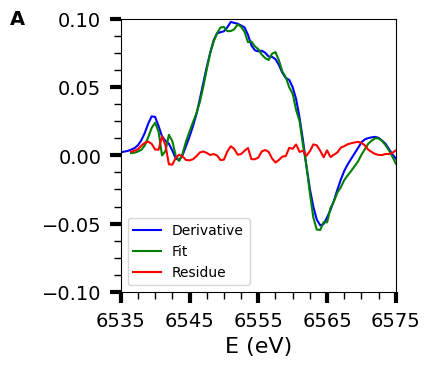  **B** |
| --- | --- |

###### **Figure S8.** Combo-fit-analysis of the XANES spectra derivatives for sample S1 beginning of the cycle (A), and for sample S2 end of the cycle (B).

**A**

######
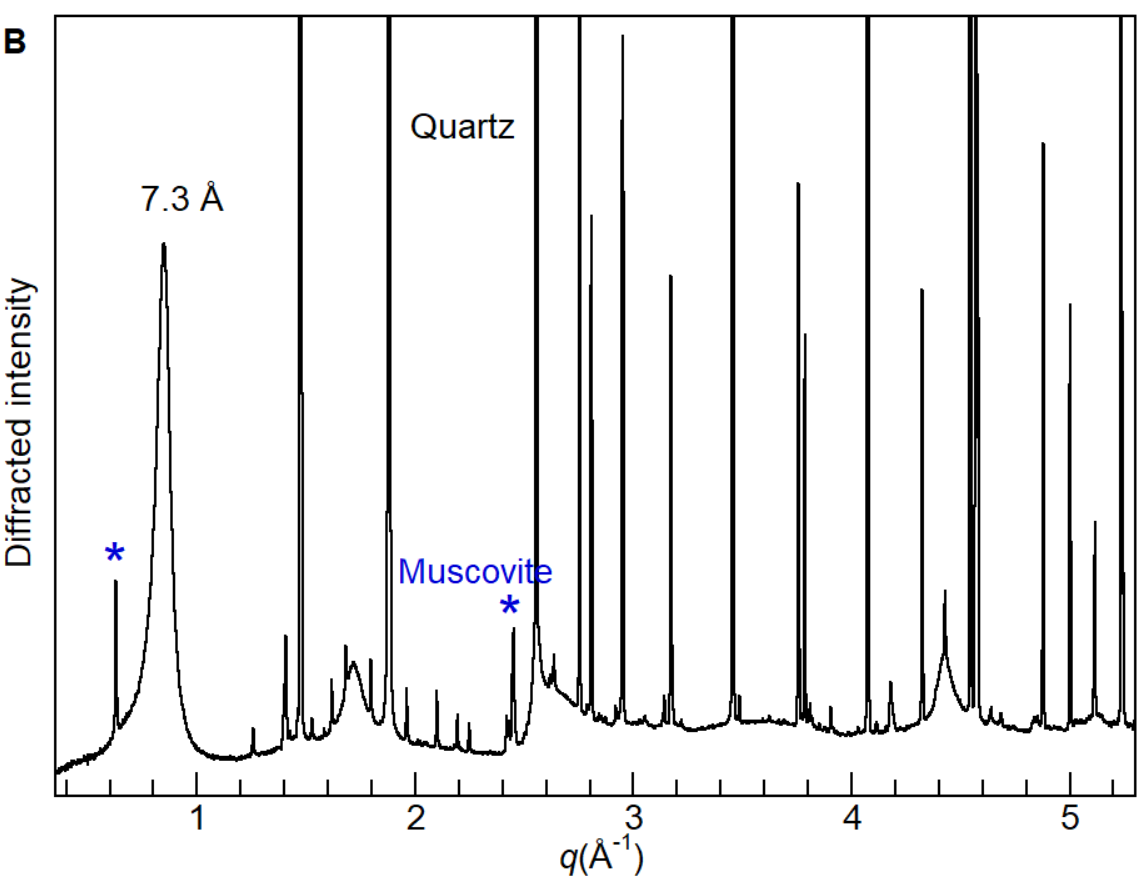


**A**


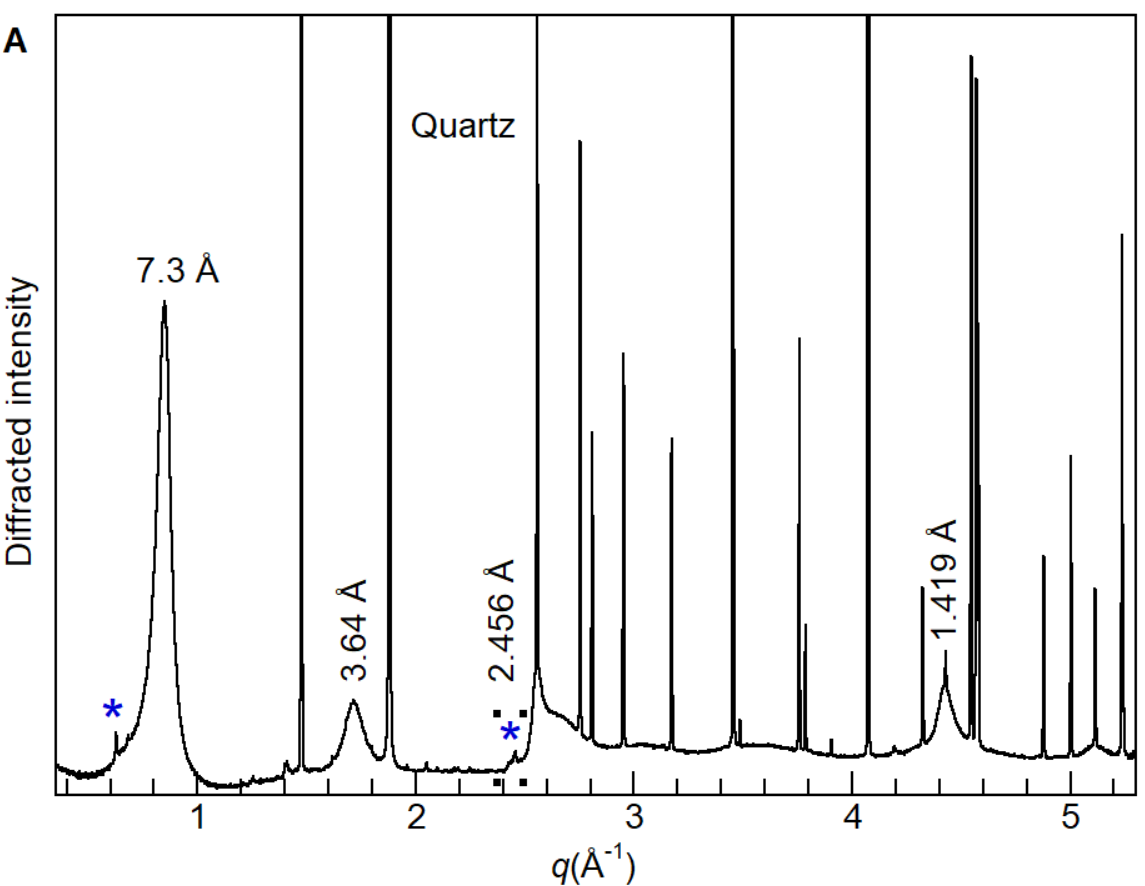


**B**

###### **Figure S9.** XRD diffractogram of the media sample at the beginning of the cycle S1 (A) and at the end S2 (B). Three phases were identified: major quartz (most intense sharp lines), major δ-MnO_2_ (broad lines), and minor muscovite 2M-1 (lower intensity sharp lines).

| 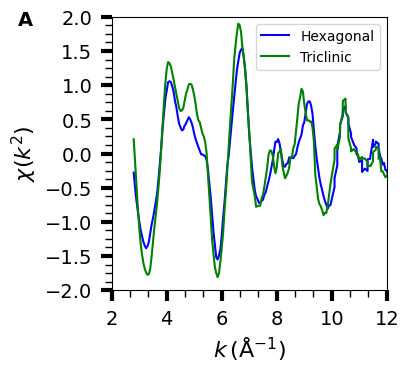 | 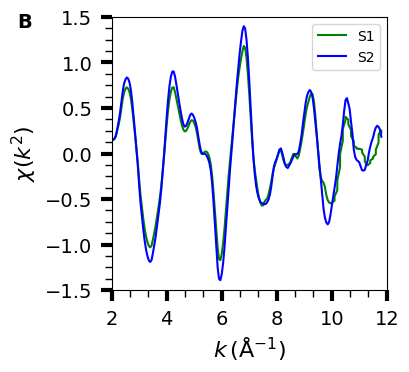 |
| --- | --- |
| 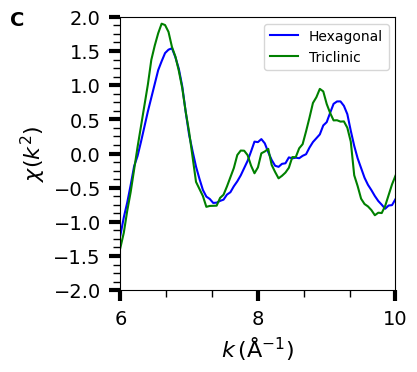 | 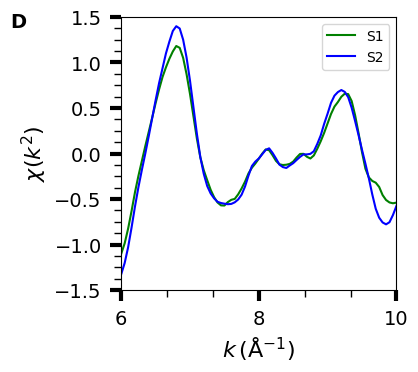 |

###### **Figure S10.** EXAFS spectra of the hexagonal and triclinic birnessite (A) of the media samples (S1 and S2) (B). Figure C and D represent a zoom of the EXAFS spectra between k = 6 and 10 Å^-1^ for the hexagonal and triclinic birnessite and for the media sample, respectively.


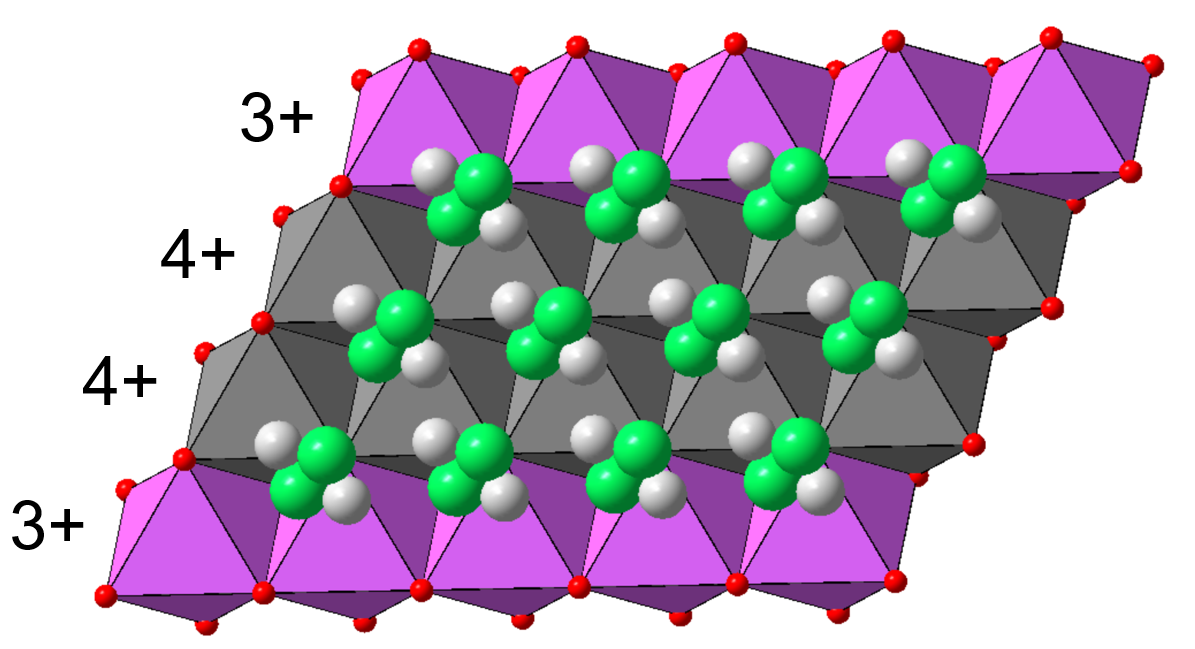


###### **Figure S11.** Structure of triclinic birnessite projected in the ab plane. The Mn(III) and Mn(IV) cations are ordered in rows (Lanson et al., 2002).


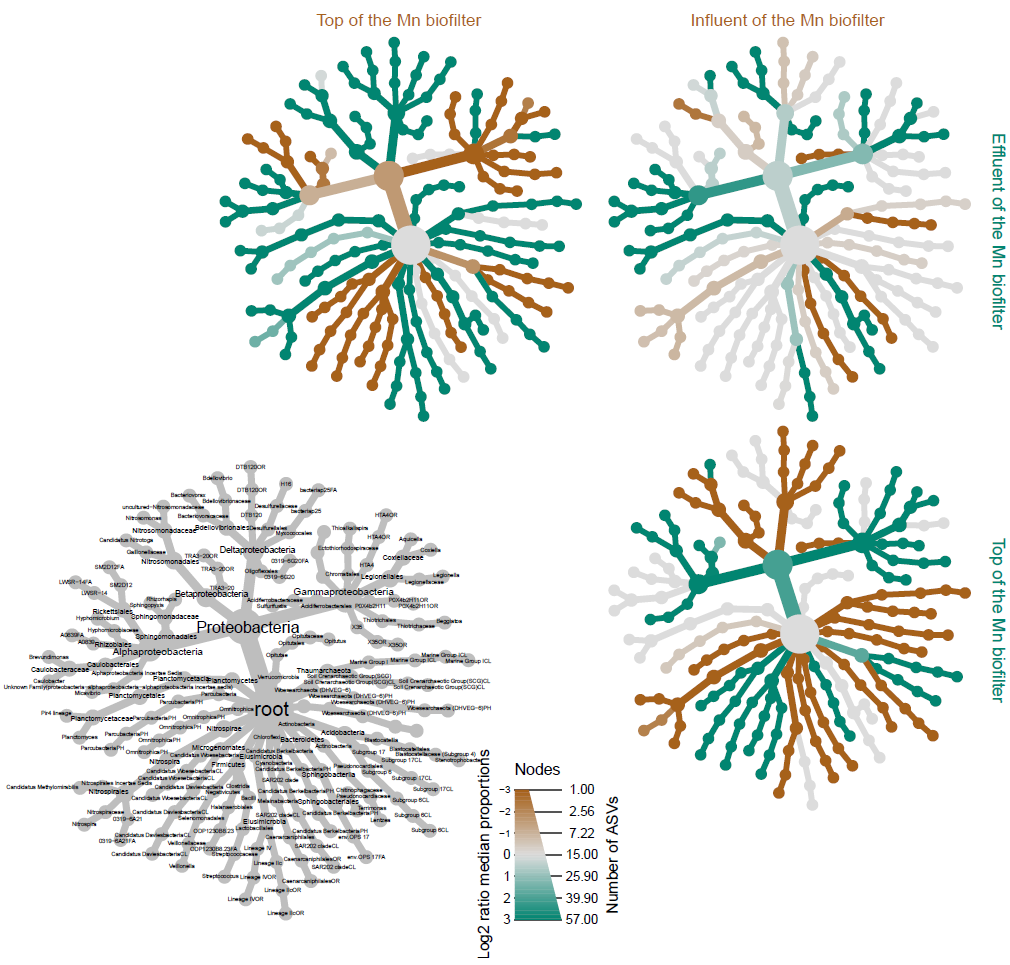


###### **Figure S12.** Heat tree presenting the taxonomic classification of microbial communities in the biofilter based on 16S rRNA sequencing. The relative abundance of key bacterial taxa is shown for the top of the biofilter, the influent, and the effluent.

| 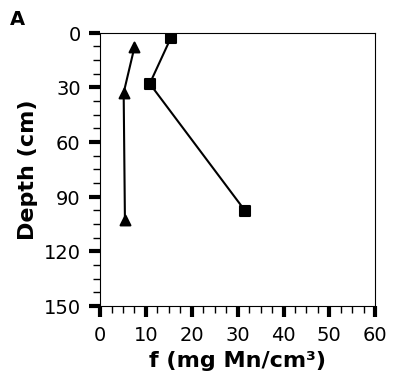 | 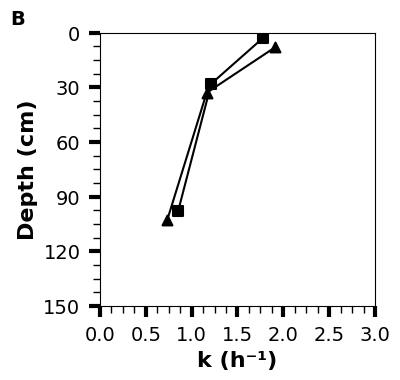 |
| --- | --- |

###### **Figure S13.** Evolution of the kinetic parameters (A. factor f, B. k) of the Mn oxidation ((■) Total biofilm, (▲) microbiological with inhibition of respiratory chain) in function of depth, at the end of the cycle.

| 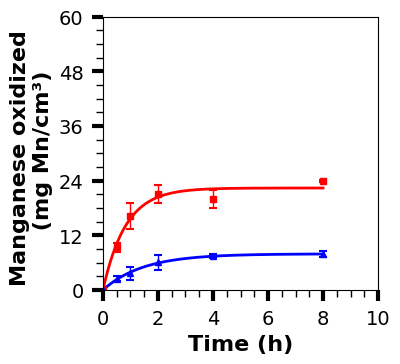  **A** | 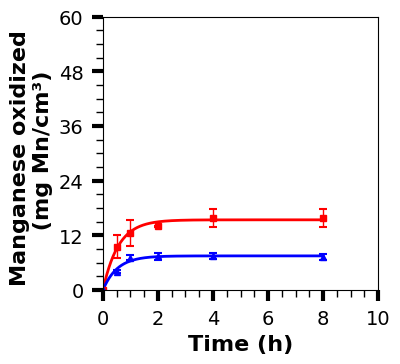  **B** |
| --- | --- |

###### ***Figure S14****. Evolution of the formation of MnOx by the extracted biofilm by the different pathways studied* ((■) Total biofilm, (▲) microbiological with inhibition of respiratory chain) *for the top (A, B) at the beginning and at the end of the cycle respectively. Pseudo-first-order kinetic model (lines) was used to model to traduce the Mn oxidation*.

**References**

Altschul, S. F., Gish, W., Miller, W., Myers, E. W., & Lipman, D. J. (1990). Basic local alignment search tool. *Journal of Molecular Biology*, *215*(3), 403-410. <https://doi.org/https://doi.org/10.1016/S0022-2836(05)80360-2>

American Public Health Association, A. W. W. A., Water Environment Federation. (2017). *Standard methods for the examination of water and wastewater* (L. L. Bridgewater, R. B. Baird, A. D. Eaton, E. W. Rice, A. American Public Health, A. American Water Works, & F. Water Environment, Eds. 23rd edition ed.). American Public Health Association.

Cerrato, J. M., Falkinham, J. O., Dietrich, A. M., Knocke, W. R., McKinney, C. W., & Pruden, A. (2010). Manganese-oxidizing and -reducing microorganisms isolated from biofilms in chlorinated drinking water systems. *Water Research*, *44*(13), 3935-3945. <https://doi.org/10.1016/j.watres.2010.04.037>

Ducret, J., & Barbeau, B. (2024). A revised digestion method to characterize manganese content in solids. *MethodsX*, *12*, 102731. <https://doi.org/10.1016/j.mex.2024.102731>

Jones, M. R., Luther, G. W., Mucci, A., & Tebo, B. M. (2019). Concentrations of reactive Mn(III)-L and MnO2 in estuarine and marine waters determined using spectrophotometry and the leuco base, leucoberbelin blue. *Talanta*, *200*, 91-99. <https://doi.org/10.1016/j.talanta.2019.03.026>

Kanaya, K., & Okayama, S. (1972). Penetration and energy-loss theory of electrons in solid targets. *Journal of Physics D: Applied Physics*, *5*(1), 43. <https://doi.org/10.1088/0022-3727/5/1/308>

Lanson, B., Drits, V. A., Feng, Q., & Manceau, A. (2002). Structure of synthetic Na-birnessite: Evidence for a triclinic one-layer unit cell. *American Mineralogist*, *87*(11-12), 1662-1671. <https://doi.org/10.2138/am-2002-11-1215>

Parada, A. E., Needham, D. M., & Fuhrman, J. A. (2016). Every base matters: assessing small subunit rRNA primers for marine microbiomes with mock communities, time series and global field samples. *Environmental Microbiology*, *18*(5), 1403-1414. <https://doi.org/10.1111/1462-2920.13023>

Servais, P., Anzil, A., & Ventresque, C. (1989). Simple Method for Determination of Biodegradable Dissolved Organic Carbon in Water. *Applied and Environmental Microbiology*, *55*, 2732-2734. <https://doi.org/10.1128/AEM.55.10.2732-2734.1989>

Tremblay, J., & Yergeau, E. (2019). Systematic processing of ribosomal RNA gene amplicon sequencing data. *GigaScience*, *8*(12), giz146. <https://doi.org/10.1093/gigascience/giz146>
